# From Smartphone Lateral Flow Immunoassay Screening to Direct MS Analysis: Development and Validation of a Semi-Quantitative Direct Analysis in Real-Time Mass Spectrometric (DART-MS) Approach to the Analysis of Deoxynivalenol

**DOI:** 10.3390/s21051861

**Published:** 2021-03-07

**Authors:** Ariadni Geballa-Koukoula, Arjen Gerssen, Michel W. F. Nielen

**Affiliations:** 1 Wageningen Food Safety Research, Wageningen University and Research, P.O. Box 230, 6700 AE Wageningen, The Netherlands; arjen.gerssen@wur.nl (A.G.); michel.nielen@wur.nl (M.W.F.N.); 2 Laboratory of Organic Chemistry, Wageningen University, Stippeneng 4, 6708 WE Wageningen, The Netherlands

**Keywords:** lateral flow immunoassay, mass spectrometry, mycotoxins, direct analysis in real time, DART, confirmatory analysis, biorecognition

## Abstract

In current food safety monitoring, lateral flow immunoassays (LFIAs) are widely used for rapid food contaminant screening. Recent advances include smartphone readouts, offering semi-quantitative analysis of LFIAs with time, location, and data transfer in case of on-site testing. Following the screening, the next step in the EU regulations is confirmation by, e.g., liquid chromatography-tandem mass spectrometry (LC-MS/MS). In this work, using direct analysis in real time ambient ionization and triple quadrupole MS/MS (DART-QqQ-MS/MS), we achieved rapid confirmation of the identity of the substance(s) causing the LFIA result. In the workflow proposed, an individual performs the (on-site) smartphone LFIA screening, and when the result is suspect, an identification LFIA (ID-LFIA) strip is developed with the same sample extract. The ID-LFIA can be dissociated and rapidly analyzed in a control laboratory with DART-QqQ-MS/MS. The ID-LFIA consists of multiple lines of monoclonal antibodies against the mycotoxin deoxynivalenol, acting as a bioaffinity trap. The ID-LFIA/DART-QqQ-MS/MS approach has been developed and validated, along with the screening smartphone LFIA, and has demonstrated its applicability by analyzing incurred and spiked samples. The developed approach has been critically compared with our previous direct electrospray ionization MS method and was found to provide highly complementary information on the total deoxynivalenol contamination in the sample.

## 1. Introduction

According to the European Union (EU) regulation, food needs to be monitored and tested to reassure the absence of contaminants [1]. Contaminants, including mycotoxins, are substances that adversely affect human health when consumed and are transferred to food during the whole food production and transportation chain [2]. Mycotoxins, such as deoxynivalenol (DON), are produced in crops contaminated by *Fusarium* sp. fungi. Because of plant metabolism (acetylation or glycosylation) DON can be converted into conjugated forms, which make analytical detection an intricate task [3]. Mycotoxins not only pose a threat to human health, but they also challenge the food industry with millions of euros in economic losses because mycotoxin contaminated food or food ingredients need to be withdrawn or destroyed before reaching the market [4]. The contaminants monitoring strategy in the EU usually consists of a two-step approach. The first step involves rapid screening assays using, for example, lateral flow immunoassays (LFIAs). These provide a positive or negative result regarding a specific contaminant’s presence or absence at a validated target level. If the screening result is positive, then confirmatory analysis should be performed with liquid or gas chromatography and (tandem) mass spectrometric detection (LC- or GC-MS or -MS/MS) [5]. In this scheme, screening and confirmation act complimentarily; screening is fast and easily performed, but it does not give any structural information on the contaminant. On the other hand, confirmation provides unequivocal detection of the contaminant at the level of interest. However, confirmation involves time-consuming chromatographic separation and elaborate sample preparation, which could be considered a disadvantage for routine analysis laboratories when many samples have to be analyzed. 

Until now, consumer’s food safety solely relies on official controls from authorities and food producers [6]. However, significant progress has been made in analytical instrumentation and food contaminant control applications, since introducing the Commission Decision 2002/657/EC, which describes the minimum performance characteristics for analytical methods to be used in screening or confirmation. Firstly, user-friendly diagnostic tests have been developed, some of which use smartphone readouts for a semi-quantitative result [7,8] and can even be used by non-experts as screening assays. The screening tests with smartphone readout provide improved sensitivity, ease of use, fast analysis, and GPS location data and time for spatiotemporal mapping of contaminations. All those offer an advantage, not only for individuals who can grasp their food safety but mostly for agri-food businesses and regulatory bodies that perform those tests. Secondly, the development of ambient ionization MS (AIMS) techniques, i.e., without time-consuming chromatographic separation, has yet to be incorporated in confirmatory analysis regulation, despite the variety of food safety applications. It is worth mentioning that the first AIMS techniques, desorption electrospray ionization (DESI) and direct analysis in real time (DART), were developed already in 2004 [9,10]. DART is an atmospheric pressure chemical ionization (APCI)-like technique that does not require any chromatographic separation, while only a low sample volume is needed. DART has been used in many applications regarding food quality and safety analysis [11,12,13], including residue analysis of drugs in bovine tissue [14], aflatoxin detection in milk [15], and mycotoxins analysis in cereals [16].

In our recently published work [17], we developed an approach for the direct MS analysis of screening LFIAs for DON with smartphone readout. At first, mycotoxins are extracted from the sample, a screening assay is performed with a smartphone readout, and the same sample extract is used to develop an identification LFIA (ID-LFIA); the latter acts as a bioaffinity trapping device. Subsequently, the analyte is retrieved from the ID-LFIA in the food control laboratory by dissociating the ID-LFIA with an alkaline methanolic solution that is directly analyzed by electrospray ionization (ESI) followed by quadrupole-orbitrap (Q-Orbitrap) MS or triple quadrupole (QqQ) MS/MS. The ID-LFIA/ESI-MS approach is to be used as a moderator for increased use in screening assays with smartphone readout, which will inevitably increase the number of samples that need to undergo confirmatory analysis to an overload of work in the food control laboratories. With the ID-LFIA/ESI-MS approach, we offered an intermediate rapid confirmatory analysis technique [17]. However, the ID-LFIA/ESI-MS approach was restricted to the negative ion (−) mode due to ion suppression caused by residues from the LFIA assay buffer residues. Consequently, ID-LFIA/(−)ESI-MS cannot detect the acetylated forms of DON (AcDON). 

In the present study, we developed a DART alternative to ionize the retrieved mycotoxins from the ID-LFIA. Because of its ionization mechanism, DART does not suffer from limitations faced at the ESI approach in the positive ion mode, such as the existence of surfactant residues and the ion suppression thereof. Moreover, using a DART ion source operating in positive ion mode made AcDON’s detection possible. The ID-LFIA/DART-MS/MS technique described in this paper has been validated in-house as a semi-quantitative confirmatory method for wheat samples spiked and incurred with the mycotoxin DON. Using the same spiked wheat samples and incurred samples, i.e., naturally contaminated crop, the preceding screening LFIA with smartphone readout has been validated as well. As such, the current ID-LFIA/DART-MS/MS approach complements the previous ID-LFIA/ESI-MS/MS approach in the detection of a DON and its conjugated forms and provides a fast direct food safety monitoring approach, thereby improving the overall efficiency and cost effectiveness in industrial and regulatory routine laboratories.

## 2. Materials and Methods

### 2.1. Chemicals and Reagents

Methanol (MeOH) of UHPLC-MS purity and ammonia solution 25% (NH_3_) were purchased from Merck (Darmstadt, Germany). Milli-Q water of 18.3 MΩ/cm conductivity was obtained with a Merck (Amsterdam, The Netherlands) water purification system. Standard stock solutions of 100 µg/mL DON, 25 µg/mL ^13^C_15_ DON, 100 µg/mL 3-AcDON, and 100 µg/mL 15-AcDON, all in ACN, were purchased from LGC standards (Wesel, Germany). 

For the in-house validation study, 21 blank wheat samples were used. Seventeen blank wheat samples previously analyzed for the absence of DON by a validated routine confirmatory LC-MS/MS method were provided in-house, and the remaining four blank samples were created by pooling eight of those samples. Portions of the blank sample extracts were spiked with a DON or 3-AcDON solution at three different target levels (TL) based on the maximum limit (ML) of 1750 μg/kg for extracted DON in unprocessed durum wheat [18] to a final level of 175 ng/mL (0.5× TL), 350 ng/mL (TL), and 525 ng/mL (1.5× TL). Additionally, wheat flour blank certified reference material (CRM) from the Joint Research Centre was purchased via LGC standards (Wesel, Germany). A contaminated corn starch sample was provided in-house. Furthermore, mouse monoclonal antibodies (mAb) for DON, clone 2, were purchased from Aokin AG (Berlin, Germany) and diluted with 1× Phosphate Buffer Saline (PBS) to a concentration of 0.3 mg/mL. The diluted mAb were used to construct the ID-LFIA as described in the previous publication [17]. In short, 15 identical lines of the diluted mAb, forming a bioaffinity trapping zone, are sprayed on top of the center of a nitrocellulose membrane (HiFlow Plus HF13502, Millipore, Carrigtwohill, Ireland) secured on a plastic backing (G & L, San Jose, CA, USA), using an XYZ3060 BioJet and AirJet instrument (Biodot Inc., Irvine, CA, USA). The nitrocellulose membrane is finally cut into strips of 5 mm width, thus creating the ID-LFIA strips, and stored in the fridge at 4 °C until further use.

For the screening assays, a smartphone-based LFIA kit, Rida Quick DON RQS Eco, including its dedicated DON extraction buffer, was obtained from r-Biopharm (Darmstadt, Germany).

### 2.2. DART-Triple Quadrupole MS/MS Conditions

The MS/MS analysis was performed on a model Xevo TQ-XS Tandem Triple Quadrupole (QqQ) MS/MS system (Waters Corporation, Milford, MA, USA) equipped with a DART SVP ion source (IonSense, Saugus, MA, USA). Optimized conditions included cone voltage 20 V, source temperature 120 °C, nanoflow nitrogen gas 0.30 bar, argon collision gas flow 0.16 mL/min, and nitrogen nebulizer gas 7.0 bar. Data were acquired in multiple reaction monitoring (MRM) mode with collision energies of 16 and 10 eV, depending on the targeted precursor and product ions (Table 1). The DART ionization source was used with helium plasma carrier gas at an optimized temperature of 350 °C and operating in positive ionization mode. DART sample introduction was performed using a 12-position metal mesh device at a rail speed of 0.6 mm/s. The total runtime for the DART-MS/MS analysis of the 12 mesh positions was less than 3.5 min. MassLynx software v4.2 (Waters) was used for data acquisition and processing of the MS data. From the chronograms acquired, area ratios of DON to that of the internal standard, ^13^C_15_ DON, were used for semi-quantitation, and ion ratio calculation of the two MRM transitions for each substance (Table 1) was used for unequivocal confirmation of the identity of each substance.

### 2.3. Sample Preparation

For all samples, the extraction protocol from the r-Biopharm smartphone-based LFIA was used: first, 5 g of grounded wheat sample was extracted by using 25 mL MilliQ water. After the addition of water, manual agitation and centrifugation at 2000× *g* for 1 min were performed to enable the solid particles’ rapid sedimentation. The resulting supernatant was used for both the smartphone-based screening LFIA and the ID-LFIA for MS analysis. For the smartphone-based screening assay, 100 μL of the sample extract was diluted with 500 μL of the assay running buffer, and from that dilution, 100 μL was used to run the smartphone-based LFIA. After 5 min, the screening result was read visually and quantified by using the smartphone r-Biopharm App. For the ID-LFIA, 100 μL of the sample extract was diluted (1:1) with assay running buffer, and the 200 μL, thus, obtained was used to run the ID-LFIA.

The protocol developed for direct MS analysis of the ID-LFIA [17] was further improved to achieve a more time-efficient procedure of retrieving the final solution from the ID-LFIA for subsequent MS analysis. First, the part of the ID-LFIA with the rectangular mAb trapping zone was cut and washed with 500 μL of MilliQ water in an Eppendorf tube under slight manual agitation for 30 s. The rectangular mAb trapping zone was then placed in an Eppendorf with 200 μL of MeOH/NH_3_ 2% *v*/*v* dissociation solution, followed by vigorous manual agitation for 1 min only. Finally, the solution was spiked at 60 ng/mL with the internal standard solution, ^13^C_15_ DON, to compensate for any remaining ion suppression and sampling irreproducibility in the subsequent DART ionization [19].

### 2.4. Initial In-House Method Validations

The smartphone-based screening LFIA method was validated in-house as a semi-quantitative screening method according to the 2002/657/EC guidelines [5], in terms of specificity, intra-, and inter-day repeatability, within-laboratory reproducibility, trueness, detection capability (CCβ), and applicability. For the specificity assessment, 21 different spiked and blank wheat samples were analyzed. Moreover, the intra- and inter-day repeatability assessment was performed by measuring seven different samples at three different spiking target levels over the course of 3 different days and assessing the calculated %RSD. Additionally, using one-way ANOVA, the within-laboratory reproducibility was calculated for the three validation days, and the trueness of the measurements, based on the theoretical concentration and comparing it with the smartphone readout result, expressed in mg/kg. To determine the CCβ, we calculated the mean response of 21 spiked samples with DON at the ML (1× TL) minus 1.64 times the standard deviation of those responses. Finally, the applicability was assessed by analyzing an incurred corn sample.

The ID-LFIA/DART-QqQ-MS/MS method was in-house validated as well as a semi-quantitative confirmatory analysis method according to the 2002/657/EC guidelines [5] for the linearity, the specificity, the intra-, and inter-day repeatability, within-laboratory reproducibility, the trueness of measurements, the decision limit (CCα) and the CCβ values, and the applicability. Blank CRM wheat extract was spiked, and the sample preparation procedure described was followed to evaluate the linearity across the TL range. The dissociation solution with the internal standard was analyzed under the optimized conditions in DART-QqQ-MS/MS. The linearity range of interest included the three spiking levels (0.5× TL, TL, and 1.5× TL) and a blank level sample (zero). For the specificity, we showed that the ID-LFIA/DART-MS/MS method could discriminate unequivocally between the analyte and other components that can be present in real samples. SPR results were used to assess the antibodies’ specificity in the ID-LFIA, and 21 different blank wheat samples were analyzed and compared with the spiked samples for the appearance of ions of interest and the respective ion ratios in the mass spectrometric method. Moreover, for the intra- and inter-day repeatability assessment, seven different samples were analyzed, and the %RSD of the measurements was calculated and assessed for three different spiking levels over the course of three different days. One-way ANOVA was used to assess the within-laboratory reproducibility for the three validation days, and the trueness was assessed using an estimate of the expected concentration (mg/kg) calculated based on each day’s calibration curve and comparing it with the theoretical concentration. To determine the CCα the value of the concentration at the ML plus 1.64 times the standard deviation of 21 blank wheat samples fortified with DON at the ML (1× TL) was calculated, and for the CCβ, the CCα value plus 1.64 times the standard deviation of 21 blank wheat samples fortified with DON at the ML (1× TL) was calculated. Finally, for the applicability, an incurred sample was measured on the final day of the validation.

## 3. Results

### 3.1. General Considerations

**Figure 1:**
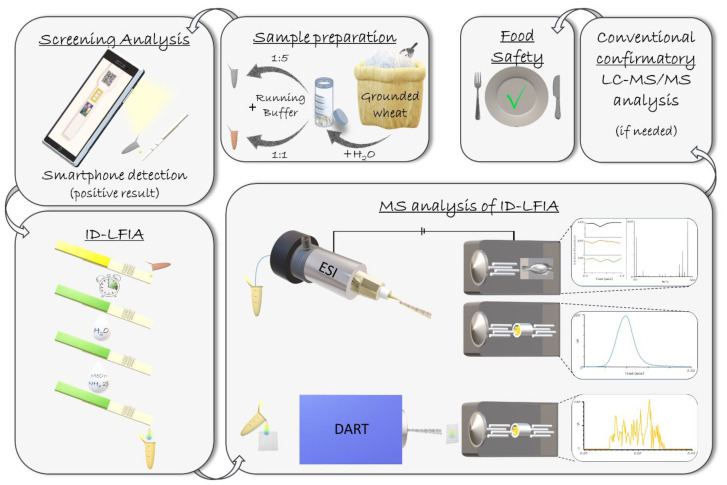
The general concept of the ID-LFIA DART (this work) and ESI [17] methods. DON is extracted from grounded wheat, and the extract is diluted with the running buffer on a 1:5 ratio. If the screening result is positive, the same sample extract is diluted with the running buffer on a 1:1 to develop the ID-LFIA. The ID-LFIA can then be further processed in the lab by washing and dissociating the bound DON and conjugates from the mAb. The final step is the rapid direct analysis by the newly developed and validated semi-quantitative DART-QqQ-MS/MS and the previously developed ESI-QqQ-MS/MS or ESI-Q-Orbitrap-MS. In case further information is needed, e.g., absolute quantitation over a different range or multitoxin analysis, conventional time-consuming LC-MS/MS analysis may be considered a follow-up.

As described in our previously published work [17], we envisage a future of smartphone-based on-site screening of food quality and safety parameters that will unavoidably increase confirmatory analysis requirements. Several smartphone-based screening methods have been developed, some of which demonstrate great citizen science potential [20,21,22]. An individual may perform on-site a rapid and user-friendly screening assay, for example, an LFIA with a smartphone readout, and if the result is positive, the same sample extract is diluted and used to develop an ID-LFIA strip, ready to be sent to the lab for subsequent confirmatory MS analysis. Following such an approach, the cause of the positive screening result at the relevant ML can be detected, and even a semi-quantitative analysis result can be achieved (Figure 1). The present ID-LFIA/DART-QqQ-MS/MS method can be used either independently or in combination with the previously developed ID-LFIA/ESI method [17], as the former indicates the presence of both DON and acetyl forms of DON, and the latter the presence of both DON and the glucoside form of DON. It is important to note that the EU legislation does not currently regulate the conjugated forms of DON yet, but revised regulations are under discussion.

### 3.2. DART-QqQ-MS/MS Method Development

**Figure 2:**
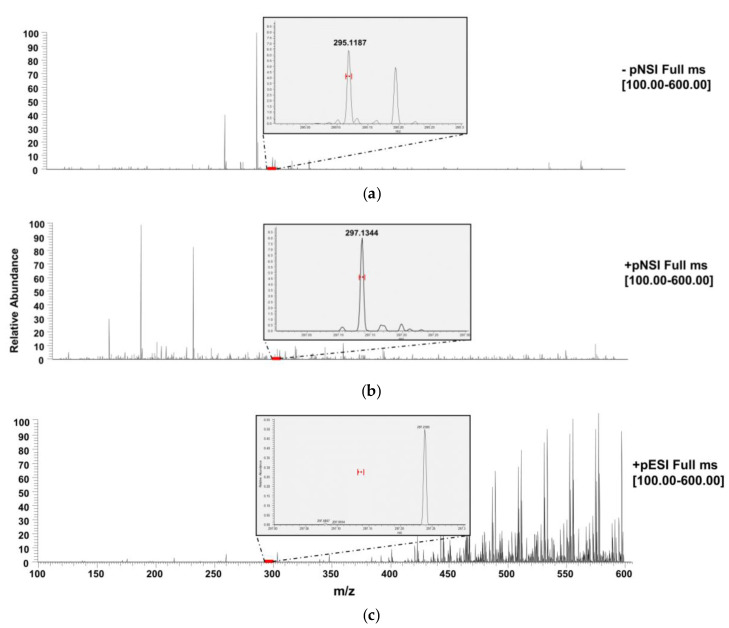
Comparison between full scan (*m*/*z* 100–600) mass spectra of a MeOH/NH_3_ 2% *v*/*v* solution of DON containing 10% *v*/*v* LFIA assay running buffer in (**a**) negative and (**b**) positive ion mode in DART-Orbitrap-MS and (**c**) positive ion mode in ESI-Orbitrap-MS. Inserted are the regions of deprotonated and protonated ions of DON, and marked in red are the expected *m*/*z* regions for the ions at 5 ppm mass accuracy.

The previous ESI method [17] was developed in negative ion mode to deal with the severe ion suppression caused by residues of assay running buffer and nitrocellulose substrate in the final ID-LFIA dissociation solution. However, because DART is an APCI-like technique known to be less prone to ion suppression, we selected the more commonly used positive ion mode for the analysis of mycotoxins [23]. In Figure 2, full scan DART-Orbitrap-MS spectra of DON in MeOH/NH_3_ 2% *v*/*v* solution, containing 10% *v*/*v* LFIA buffer, can be seen in negative and positive ion mode. Both modes can detect DON using the DART ion source, contrary to the previously published ESI mode [17] that showed high interferences and ion suppression in positive ion mode. Although under ambient conditions, positive ion mode is in general characterized by a higher background compared to negative ion mode DART, the protonated ion of DON at *m*/*z* 297.1344 at 5 ppm mass accuracy detection window, can be easily detected in DART, whereas in (+)ESI ionization, it is not (Figure 2). Moreover, the positive ion mode gave us the ability to measure AcDON, which was not possible in the previous negative ion ID-LFIA/ESI-MS approach [17]. Preliminary DART experiments performed in a standard solution of 1 μg/mL in MeOH/NH_3_ 2% *v*/*v* and using Orbitrap-MS in full scan negative ion mode suggested an ability to detect DON3G as well through its deprotonated and its chlorine and formic acid adduct ions (Figure S1 in Supplementary Material). Nevertheless, in agreement with previous DART attempts [16], the DART’s sensitivity turned out to be inadequate for DON3G detection when trying to reach more food-safety-relevant levels.

To perform the optimization of the QqQ MS/MS fragmentation conditions, the ion transitions’ intensities were tested in MRM mode, following a stepwise increase in collision energy. DART is an open ion source, so we could not merely assess the fragment ions from a product ion scan because of ambient interferences biasing the optimum collision energy. To give an example, for the DON *m*/*z* 297 > 249 MRM transition: the suggested optimum value of 30 eV was unable to differentiate between blank and spiked samples. In contrast, the second-best intense value in the absolute intensity signal versus the collision energy plot did successfully discriminate between spiked and blank samples (Figure S2 in Supplementary Material). Note that because of the lack of chromatographic separation and the production of common fragment ions from 3-AcDON and 15-AcDON, we cannot discriminate between them in direct MS methods such as DART; instead, the ID-LFIA/DART-QqQ-MS/MS method detects apart from DON the total AcDONs. All the other MS/MS conditions were the same as in the previously developed ESI-QqQ-MS/MS method [17], except for the cone voltage that was adjusted to 20 V, because it yielded more reproducible results in DART-QqQ-MS/MS.

Next, different compositions of ID-LFIA dissociation solutions were tested for DON in DART-MS/MS, such as MeOH or ACN with an organic modifier of HCOOH or NH_3_ 2% *v*/*v*. As with the previously developed ESI approach [17], MeOH/NH_3_ 2% *v*/*v* showed the highest signal intensity for both DON and acetylated form of DON (Figure S3 in Supplementary Material). 

**Figure 3:**
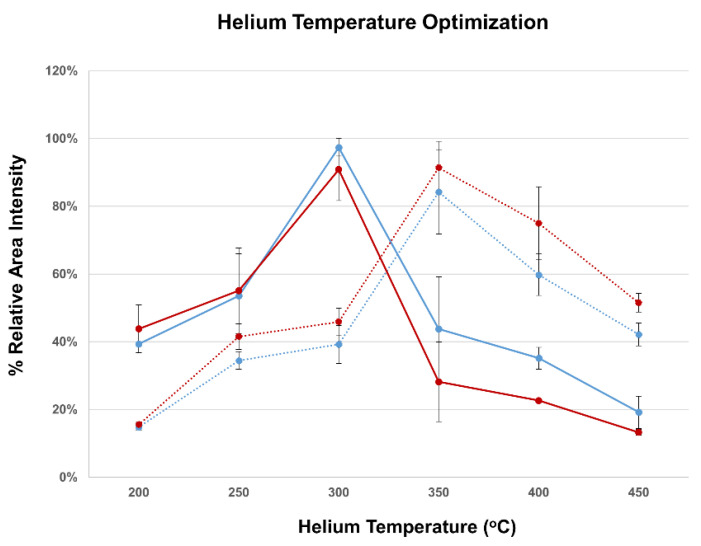
DART helium gas temperature optimization in QqQ-MS/MS for DON (blue) and 3-AcDON (red) 60 ng/mL, in MeOH/NH_3_ 2% *v*/*v* (solid line) and MeOH/NH_3_ 2% *v*/*v* exposed to the ID-LFIA substrate (dotted line). Measurements were performed in duplicate by MRM monitoring of the ion transitions specified in Table 1. The standard deviation of the duplicate measurement is shown by the error bars. The results are presented as the relative (%) area intensity, i.e., the ratio of the absolute area intensity divided by the highest absolute area intensity observed for each substance in each solution.

Finally, optimization of the settings for the DART helium carrier gas was performed. Differences in DART gas temperature may lead to significant changes in the desorption/ionization efficiency and the method’s sensitivity [24,25,26]. When the solution used for optimization was only MeOH/NH_3_ 2% *v*/*v*, spiked with DON or 3-AcDON at 60 ng/mL, the optimum DART temperature was found to be 300 °C for both analytes. In contrast, when a matrix-matched solution from ID-LFIA developed with blank assay buffer and washed, and the final dissociation solution MeOH/NH_3_ 2% *v*/*v* was spiked, the optimum DART temperature was 350 °C. More importantly, the signal intensity doubles when the temperature increases from 300 to 350 °C, which underlines the importance of carrier gas settings optimization (Figure 3).

### 3.3. Initial In-House Method Validations

#### 3.3.1. Validation of Screening LFIA with Smartphone Readout

**Figure 4:**
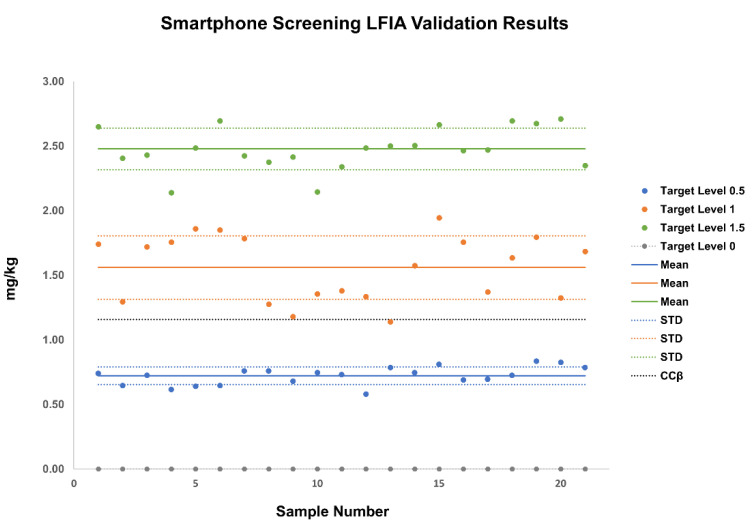
Data analysis of 21 blank wheat samples, and spiked versions thereof, at three target levels, demonstrating distinct differentiation between them in the screening LFIA. Quantification with the use of the smartphone app.

The specificity of the screening LFIA with smartphone readout was assessed by analyzing 21 blank wheat and spiked samples thereof. Based on the results, there was sufficient discrimination between blank and spiked samples from the 0.5× TL level onwards (Figure 4 and Table 2).

Moreover, the repeatability, both intra- and inter-day were assessed for the three validation levels. The intra-day repeatability results expressed as the %RSD were 7.9, 10.4, and 6.9% at 0.5× TL, 8.9, 10.1, and 4.9% at TL, and 7.4, 12.7, and 5.2% at the 1.5× TL levels, respectively. The inter-day repeatability expressed by the %RSD was 9.4, 15.8, and 6.5% at 0.5×, 1 ×, and 1.5× TL, respectively. All RSD% values for the repeatability assessed were lower than 20% and within the acceptance range.

The %RSD to assess the within-laboratory reproducibility was calculated using one-way ANOVA for each TL. For the smartphone screening LFIA, the results were 10.0, 17.6, and 6.8% at 0.5×, 1 ×, and 1.5× TL, respectively. According to the quantitative performance criteria for the validation of substances with an established ML, %RSD should be not greater than the corresponding reproducibility at the 0.5× TL [5], which indicated that the calculated value for the 1× TL was not within the acceptance range, and consequently, the smartphone-based LFIA should be considered as a semi-quantitative screening method.

For the trueness assessment of the smartphone screening LFIA, the theoretical concentration at each spiking level was compared to that of the smartphone readout result, expressed in mg/kg. The calculated trueness values were 83, 89, and 94% at the 0.5×, 1×, and 1.5× TL, respectively, and within the acceptance range of 80 to 110% listed in the regulation [5].

Finally, the compliance of the method versus the performance criteria was assessed using the CCβ value. The CCβ was calculated at 1156 μg/kg for 21 DON-spiked wheat samples at the ML level. This indicates a 5% probability that the screening result is falsely characterized as compliant sample (screening result below the ML), if the result of the screening assay is less than 1156 μg/kg (false negative). For the 21 DON-spiked wheat samples analyzed at the 1× TL, only sample 13 resulted in a readout of 1140 μg/kg, i.e., below the CCβ, meaning that the smartphone LFIA screening passed the performance criteria for a screening method and has only a 5% probability that the result is falsely classified as compliant.

#### 3.3.2. Validation of ID-LFIA/DART-QqQ-MS/MS

The linear range of the recovered DON from the ID-LFIA/DART-QqQ-MS/MS was found to be 0–525 ng/mL, corresponding to a range of 0 to 1.5× the ML of 1750 μg/kg of DON in unprocessed durum wheat with a regression coefficient of 0.987, demonstrating semi-quantitative performance in the relevant range (Figure S4 in Supplementary Material). 

**Figure 5:**
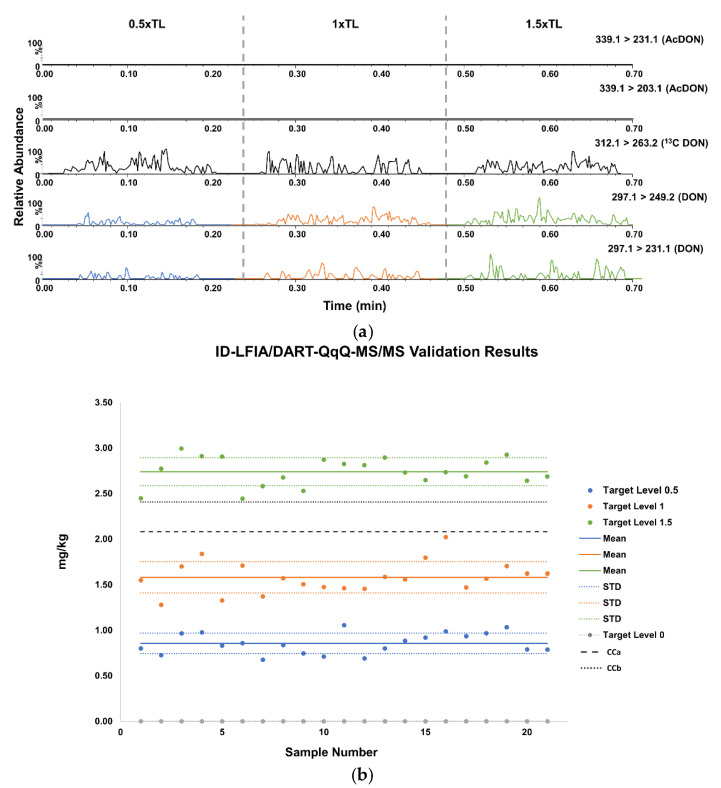
(**a**). Representative chronograms for DON-spiked wheat samples used to develop the ID-LFIA, retrieved in dissociation solution and analyzed by DART-QqQ-MS/MS, demonstrating the three distinct spiking levels in blue 0.5× TL, orange 1× TL, and green 1.5× TL. ^13^C_15_ DON is added as an internal standard before the MS/MS analysis. (**b**). Data analysis of 21 blank wheat samples, and spiked versions thereof in ID-LFIA/DART-QqQ-MS/MS, at three target level concentrations in blue 0.5× TL, orange 1× TL, and green 1.5× TL.

The ID-LFIA/DART-QqQ-MS/MS analysis’ specificity is justified based on the use of specific antibodies that isolate the analyte of interest on the ID-LFIA. For assessment of the specificity of the antibodies in the ID-LFIA, SPR measurements were performed. The results showed no interaction of the mAb with other mycotoxins produced by *Fusarium* species and likely to be present in wheat, such as T-2 toxin, fumonisin B2, and zearalenone [27]. However, the mAb demonstrated binding with nivalenol and the conjugated forms of DON such as AcDON and DON3G (Figure S5 in Supplementary Material). Apart from the intrinsic and prominent biorecognition characteristic that the ID-LFIA adds to the overall specificity, the MS analysis that follows can differentiate between DON and AcDONs. The selected MRM transitions monitored for each substance (Table 1), as well as the respective ion ratios (Table 2), demonstrated robustness throughout the analysis of all samples and enabled the confirmation of the identity. More specifically, the mean ion ratio for the MRM transitions of DON was 0.29 (29%) with a %RSD of 9.2% for all the 21 spiked samples analyzed. This ratio is identical to the 0.29 ion ratio measured for DON standard solutions in MeOH/NH_3_ 2% *v*/*v* and in MeOH/NH_3_ 2% *v*/*v* exposed to the ID-LFIA substrate. As a result, neither the wheat sample matrix nor the ID-LFIA substrate affect the fragmentation of DON, and the ion ratio tolerance limit of the spiked wheat samples analyzed complies with the regulatory requirement of ±25% for earning three identification points (IPs) [5]. The method specificity was assessed through the analysis of blank samples in MS. The 21 blank samples analyzed demonstrated no signal for the selected ion transitions, and the method is, thus, characterized by sufficient specificity to differentiate between blank and spiked real samples (Figure 5 and Table 2).

Moreover, the repeatability, both intra- and inter-day were assessed for the three validation levels. For the ID-LFIA/DART-QqQ-MS/MS, the intra-day repeatability results expressed as the %RSD was 14.1, 13.9, and 8.7% at 0.5× TL, 16.9, 3.7, and 4.5% at TL, and 9.6, 14.5, and 3.6% at the 1.5× TL level, for day one, two, and three, respectively. The inter-day repeatability expressed by the %RSD was 16.9, 14.9, and 10.2% for the 0.5×, 1×, and 1.5× TL, respectively, again underlining the performance of the developed ID-LFIA/DART-QqQ-MS/MS method, thus comparing nicely with the semi-quantitative smartphone screening assay. All RSD% values for the validation parameters assessed were lower than 20% and within the acceptance range.

Furthermore, to assess the within-laboratory reproducibility, the %RSD was calculated using one-way ANOVA, for each TL calculated at 18.4, 16.0, and 11.6% at 0.5×, 1×, and 1.5× TL, respectively. According to the regulation, the %RSD should be not greater than the corresponding reproducibility at the 0.5× TL for substances with defined ML [5], which means that all measurements were within the acceptance range.

Moreover, the compliance of the method versus the performance criteria was assessed using the CCα and CCβ values. The CCα was calculated at 2078 μg/kg and the CCβ at 2406 μg/kg for 21 DON-spiked wheat samples at the ML level. For the samples at 1.5× TL, all measurements were above the CCβ, so the samples contain DON above the ML level with a probability of 95% (and 5% of false positive). 

Finally, for the ID-LFIA/DART-QqQ-MS/MS, an estimate of the expected mg/kg concentration was calculated, based on each day’s calibration curve, and compared with the theoretical concentration at each spiking level to calculate the trueness of the measurements. The calculated trueness was 101, 91, and 104% for the 0.5×, 1×, and 1.5× TL, respectively. All measurements were within the acceptance range of 80 to 110% listed in the regulation [5]. Therefore, the developed ID-LFIA/DART-QqQ-MS/MS has been successfully validated as a semi-quantitative confirmatory method. 

### 3.4. Analysis of Real Samples

**Figure 6:**
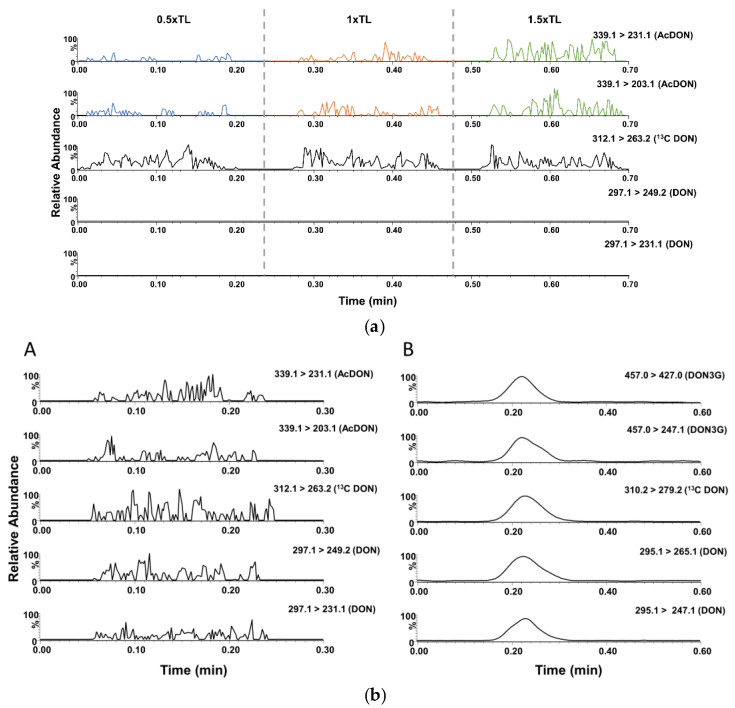
(**a**). Chronogram of AcDON-spiked wheat sample used to develop the ID-LFIA and retrieved in dissociation solution analyzed by DART-QqQ-MS/MS, demonstrating the three distinct TL in blue 0.5× TL, orange 1× TL, and green 1.5× TL. ^13^C_15_ DON is added as an internal standard before the MS analysis. (**b**). Chronogram of the incurred corn starch sample used to develop the ID-LFIA and retrieved in dissociation solution analyzed in **A**. (+)DART-QqQ-MS/MS and **B**. (−)ESI-QqQ-MS/MS in flow injection analysis mode. ^13^C_15_ DON is added as an internal standard prior to MS analysis.

The applicability of the method is further demonstrated by the analysis of additional spiked and incurred samples. AcDON-spiked samples were analyzed at the same spiking levels as the DON validation TL. The presence of AcDON was confirmed by the presence of the selected MRM ion transitions and the respective ion ratio compared with a standard spiked MeOH/NH_3_ 2% *v*/*v* solution. The mean ion ratio for AcDON in the spiked wheat samples analyzed was 0.71 (71%) with an %RSD of 12%, while the ion ratio of the standard spiked MeOH/NH_3_ 2% *v*/*v* solution was calculated at 0.76 (76%). As a result, the ion ratio tolerance limit of the AcDON-spiked wheat sample analyzed was within the regulatory requirements of ±20%, and three IPs were obtained [5]. The response factor of AcDON, i.e., the area ratio of AcDON and the internal standard ^13^C_15_ DON, was used to differentiate between the three different spiking levels. The response factor for each spiking level clearly differentiates, as no overlapping can be observed between each mean response factor value (± standard deviation). This adds to the semi-quantitative attributes of the ID-LFIA/DART-QqQ-MS/MS approach (Figure 6a and Table 3).

Moreover, a contaminated corn starch sample was analyzed, both with the ID-LFIA/ESI-QqQ-MS/MS [17] and the ID-LFIA/DART-QqQ-MS/MS approach. The smartphone-based LFIA screening revealed a highly contaminated sample (Table 3). The ID-LFIA-MS/MS results also indicated a high contaminated (>>1.5× TL) sample and were highly complementary, as the ESI method detected the presence of DON and DON3G, and the DART method detected DON and AcDON (Figure 6b). In accordance with the workflow depicted in Figure 1, these results were also confirmed by an accredited LC-MS/MS confirmatory analysis method, demonstrating high contamination quantified as 13 mg/kg DON, 2.1 mg/kg 15-AcDON, 0.13 mg/kg 3-AcDON, and 0.52 mg/kg DON3G.

## 4. Discussion

In the modern world, people are highly dependent on their phones. Smartphones have endless possibilities, from monitoring our biorhythm to navigating us with accurate mapping [28]. One of the emerging possibilities of smartphone use is monitoring food quality and safety [29]. Soon, smartphone-based screening diagnostics in food safety analysis will not be speculation but reality, and the high demands for confirmatory analysis will overrun the routine analysis laboratories. Combining two techniques, screening and confirmatory, and using them to concentrate solely on the positive of each one of them, with the so-called hyphenated techniques’ approach, is the aim of many studies [30]. The ID-LFIA/DART-QqQ-MS/MS approach presented in this work combines two techniques to improve food safety testing schemes. The developed and validated approach could be considered for application to any other low molecular weight contaminant for on-site screening and confirmation in future food control frameworks, provided antibodies are available and the contaminant has a sufficient ionization efficiency.

At first, the method was optimized, and afterward, it was validated according to the 2002/657/EC guidelines [5]. During the method optimization, we observed the significance of choosing the right temperature for the helium carrier gas and the collision energy in DART operation and AIMS in general. Contrary to our group’s previously published work, the ID-LFIA/ESI-MS approach [17], the DART approach can deal more efficiently with the ion suppression caused by the LFIA buffer residues. Additionally, the DART-QqQ-MS/MS approach was validated as a semi-quantitative confirmatory method for DON in a limited but relevant concentration range around the regulatory limit. The number of lines of DON mAb enable the binding of a limited amount of DON, which allows for a semi-quantitative approach around the relevant ML, but not for absolute quantitation over a large linearity range. The robust ion ratios monitored showed a well-performing method for the detection of DON and its acetyl forms. For the unambiguous confirmation of the identity of the target analytes selected, MRM transitions were monitored, and their respective ion ratios were monitored with robustness for all spiked and incurred samples. According to the confirmatory analysis performance criteria for group B substances (i.e., veterinary drugs and contaminants including mycotoxins), three identification points (IPs) are required for substances’ identification [5]. Even without additional IPs from a chromatographic separation, with QqQ, two product ions from the MRM ion monitoring yield sufficient three IPs for substances’ identification. In the future revised regulation, additional IPs might be granted to this approach because of the immunochromatographic properties of the ID-LFIA. Moreover, the accompanied smartphone LFIA screening was validated as well, leading to a wholly validated approach for the detection of DON: smartphone screening LFIA followed by ID-LFIA/DART-QqQ-MS/MS analysis. 

We acknowledge that our validated method is not the only LFIA or DART method published so far to detect DON. However, our method combines both LFIA and DART analysis, and it addresses several issues not coped with before. For instance, the screening (smartphone) LFIA methods published [31,32] cannot discriminate between the DON and its conjugated forms that yield the positive result. On the other hand, the DART-MS method published [16], even though it measures a higher number of mycotoxins, it does not yield enough IPs to be classified as a confirmatory approach according to the regulation, because of the full scan high-resolution detection of only a single *m*/*z* value for each mycotoxin. Finally, the conventional GC- MS or LC- MS/MS methods developed [33,34,35,36] require time-consuming sample preparation/pretreatment including derivatization in case of GC analysis and chromatographic separation, which can reach over 15 min per sample; and in case of LC- analysis, it consumes large amounts of solvents.

With the validated smartphone LFIA followed by ID-LFIA/DART-QqQ-MS/MS approach, we can focus on each technique’s positives and escape possible limitations. For this reason, the inability of the LFIA to discriminate the reason behind the positive result can be addressed with the MS analysis that follows, and the full scan monitoring to detect the mycotoxin under investigation can be addressed by the ID-LFIA, isolating only the mycotoxin group of interest. Other characteristics that make our method suitable for future use by routine confirmatory analysis laboratories and suitable to be incorporated in future food safety monitoring are its increased throughput, low sample and solvent volumes used, instrument operating time and minimal sample preparation needed, as well as its robustness on the monitoring of the relevant ion transitions.

## Figures and Tables

**Table 1:** MRM ion transitions for DON, ^13^C_15_ DON, and AcDON in triple quadrupole MS/MS and respective optimized collision energies.

Analyte	MRM Ion Transitions (*m*/*z*) (Positive Ion Mode)	Collision Energy (eV)
DON	297.1 > 249.2	10
	297.1 > 231.1	16
^13^C_15_ DON	312.1 > 263.2	10
	312.1 > 245.1	16
AcDON	339.1 > 231.1	10
	339.1 > 203.1	16

**Table 2:** Results from ID-LFIA/DART-QqQ-MS/MS analysis and the respective LFIA smartphone screening of 21 validation blank and spiked wheat samples at three different target levels and performed on three different days.

Day	Sample	0× TL		0.5× TL			1× TL			1.5× TL		
		ID-LFIA/DART-QqQ-MS/MS Response Factor Versus ^13^C_15_ DON ± SD	LFIA Smartphone Screening Result (mg/kg, Mean Response ± SD)	ID-LFIA/DART-QqQ-MS/MS		LFIA Smartphone Screening Result (mg/kg, Mean Response ± SD)	ID-LFIA/DART-QqQ-MS/MS		LFIA Smartphone Screening Result (mg/kg, Mean Response ± SD)	ID-LFIA/DART-QqQ-MS/MS		LFIA Smartphone Screening Result (mg/kg, Mean Response ± SD)
				Mean Ion Area Ratio of DON ± SD	Expected mg/kg ± SD		Mean Ion Area Ratio of DON ± SD	Expected mg/kg ± SD		Mean Ion Area Ratio of DON ± SD	Expected mg/kg ± SD	
1	1	0	<0.25	0.30 (±0.03)	0.80 (±0.01)	0.74 (±0.01)	0.27 (±0.03)	1.55 (±0.06)	1.74 (±0.01)	0.29 (±0.05)	2.40 (±0.02)	2.65 (±0.00)
	2	0	<0.25	0.30 (±0.00)	0.73 (±0.01)	0.65 (±0.02)	0.32 (±0.01)	1.28 (±0.03)	1.30 (±0.01)	0.30 (±0.03)	2.77 (±0.01)	2.41 (±0.01)
	3	0	<0.25	0.29 (±0.02)	0.97 (±0.06)	0.73 (±0.01)	0.28 (±0.01)	1.70 (±0.03)	1.72 (±0.02)	0.32 (±0.01)	2.99 (±0.03)	2.43 (±0.02)
	4	0	<0.25	0.28 (±0.04)	0.98 (±0.01)	0.62 (±0.01)	0.34 (±0.00)	1.84 (±0.04)	1.76 (±0.01)	0.28 (±0.00)	2.91 (±0.11)	2.14 (±0.03)
	5	0	<0.25	0.28 (±0.01)	0.83 (±0.03)	0.64 (±0.00)	0.30 (±0.02)	1.33 (±0.01)	1.86 (±0.02)	0.27 (±0.00)	2.91 (±0.12)	2.49 (±0.03)
	6	0	<0.25	0.32 (±0.01)	0.86 (±0.02)	0.65 (±0.01)	0.23 (±0.00)	1.71 (±0.04)	1.85 (±0.03)	0.32 (±0.02)	2.42 (±0.00)	2.70 (±003)
	7	0	<0.25	0.31 (±0.02)	0.68 (±0.02)	0.76 (±0.00)	0.31 (±0.02)	1.37 (±0.00)	1.79 (±0.02)	0.30 (±0.01)	2.58 (±0.06)	2.43 (±0.01)
2	8	0	<0.25	0.34 (±0.00)	0.84 (±0.02)	0.76 (±0.00)	0.31 (±0.02)	1.57 (±0.02)	1.28 (±0.01)	0.31 (±0.02)	2.98 (±0.01)	2.38 (±0.02)
	9	0	<0.25	0.34 (±0.01)	0.75 (±0.02)	0.68 (±0.01)	0.25 (±0.00)	1.51 (±0.03)	1.18 (±0.01)	0.29 (±0.03)	2.53 (±0.01)	2.42 (±0.00)
	10	0	<0.25	0.26 (±0.01)	0.71 (±0.03)	0.75 (±0.01)	0.27 (±0.02)	1.47 (±0.06)	1.36 (±0.01)	0.29 (±0.02)	2.87 (±0.07)	2.15 (±0.02)
	11	0	<0.25	0.31 (±0.02)	1.06 (±0.01)	0.73 (±0.00)	0.28 (±0.05)	1.46 (±0.02)	1.38 (±0.00)	0.28 (±0.04)	2.82 (±0.00)	2.34 (±0.07)
	12	0	<0.25	0.31 (±0.01)	0.69 (±0.00)	0.58 (±0.01)	0.28 (±0.01)	1.46 (±0.00)	1.34 (±0.01)	0.33 (±0.02)	2.81 (±0.01)	2.49 (±0.05)
	13	0	<0.25	0.30 (±0.04)	0.80 (±0.01)	0.79 (±0.01)	0.30 (±0.02)	1.59 (±0.01)	1.14 (±0.01)	0.31 (±0.04)	2.90 (±0.05)	2.50 (±0.00)
	14	0	<0.25	0.26 (±0.01)	0.88 (±0.00)	0.75 (±0.01)	0.24 (±0.00)	1.56 (±0.00)	1.58 (±0.03)	0.27 (±0.01)	2.73 (±0.00)	2.51 (±0.00)
3	15	0	<0.25	0.31 (±0.03)	0.92 (±0.02)	0.81 (±0.00)	0.26 (±0.03)	1.80 (±0.07)	1.95 (±0.02)	0.29 (±0.02)	2.65 (±0.01)	2.67 (±0.02)
	16	0	<0.25	0.33 (±0.02)	0.99 (±0.03)	0.69 (±0.01)	0.25 (±0.02)	2.12 (±0.02)	1.76 (±0.02)	0.29 (±0.05)	2.73 (±0.03)	2.47 (±0.03)
	17	0	<0.25	0.33 (±0.01)	0.94 (±0.01)	0.70 (±0.02)	0.31 (±0.03)	1.47 (±0.03)	1.37 (±0.01)	0.29 (±0.01)	2.69 (±0.04)	2.47 (±0.01)
	18	0	<0.25	0.30 (±0.04)	0.97 (±0.03)	0.73 (±0.05)	0.25 (±0.00)	1.57 (±0.01)	1.64 (±0.02)	0.26 (±0.01)	2.84 (±0.07)	2.70 (±0.03)
	19	0	<0.25	0.27 (±0.02)	1.03 (±0.01)	0.84 (±0.04)	0.30 (±0.03)	1.70 (±0.02)	1.80 (±0.01)	0.31 (±0.01)	2.92 (±0.07)	2.68 (±0.01)
	20	0	<0.25	0.29 (±0.00)	0.79 (±0.02)	0.83 (±0.06)	0.26 (±0.02)	1.62 (±0.01)	1.33 (±0.01)	0.34 (±0.00)	2.64 (±0.01)	2.71 (±0.03)
	21	0	<0.25	0.32 (±0.00)	0.79 (±0.03)	0.79 (±0.01)	0.25 (±0.00)	1.62 (±0.06)	1.69 (±0.05)	0.32 (±0.01)	2.69 (±0.05)	2.35 (±0.25)

Conditions: a duplicate sampling of the same final ID-LFIA dissociation solution in DART-QqQ-MS/MS. SD is the standard deviation of the duplicate measurements. The screening result was performed with the smartphone r-Biopharm LFIA, and the quantification of the screening was done with the associated smartphone app. SD is the standard deviation of duplicate reading of the same LFIA. The expected mg/kg is the concentration estimate calculated from each day’s calibration curve based on the response factor (mean ratio of DON MRM product ion at m/z 249.2 to the ^13^C_15_ DON MRM product ion at *m*/*z* 263.2). The DON ion ratio is the mean ratio of the absolute area of the DON MRM product ion at *m*/*z* 231.1 to the area of the ion at *m*/*z* 249.2.

**Table 3:** Results from ID-LFIA/DART-QqQ-MS/MS analysis and the respective LFIA smartphone screening of AcDON-spiked wheat samples at three target levels and the analysis of an incurred corn starch sample.

	TL	ID-LFIA/DART-QqQ-MS/MS				LFIA Smartphone Screening Result (mg/kg, Mean Response ± SD)
		Mean Ion Area Ratio of DON ± SD	Response Factor ± SD	Mean Ion Area Ratio of AcDON ± SD	Response Factor ± SD	
AcDON-spiked wheat	0.5	-	-	0.73 (±0.06)	0.09 (±0.01)	0.64 (±0.01)
	1	-	-	0.76 (±0.03)	0.16 (±0.02)	1.87 (±0.04)
	1.5	-	-	0.65 (±0.02)	0.31 (±0.01)	2.62 (±0.03)
Contaminated corn starch		0.32 (±0.09)	0.82 (±0.08)	0.71 (±0.08)	0.45 (±0.05)	12.76 (±0.07)

Conditions: duplicate sampling of the same final ID-LFIA dissociation solution in DART-QqQ-MS/MS. SD is the standard deviation of the duplicate measurements. The screening result was performed with the r-Biopharm LFIA, and the quantification was done with the associated smartphone app. SD is the standard deviation of duplicate reading of the same LFIA. The response factor for DON is the mean area ratio of the MRM product ion at *m*/*z* 249.2 and the ^13^C_15_ DON ion at *m*/*z* 263.2, and for AcDON the ratio of the MRM product ion at *m*/*z* 231.1 and the ^13^C_15_ DON ion at m/z 263.2. The DON ion ratio is the ratio of the absolute area of the DON MRM product ion at *m*/*z* 231.1 to the area of the ion at *m*/*z* 249.2 and for AcDON the ratio of the MRM product ions at *m*/*z* 203.1 and *m*/*z* 231.1.

## Data Availability

Data are contained within the article.

## Supplementary Materials

The following are available online at https://www.mdpi.com/1424-8220/21/5/1861/s1, Figure S1: Ionization in DART-Orbitrap MS negative ion mode, Figure S2: Fragmentation optimization of DON, AcDON, and ^13^C_15_ DON, Figure S3: Chronograms of positive ionization DART-MS/MS of different solutions, Figure S4. Linearity curve of DON retrieved from ID-LFIA-DART-QqQ-MS/MS analysis of wheat spiked samples, Figure S5: Overlay SPR sensorgrams from the competitive inhibition measurements of *Fumonisin* sp. toxins.
