# Supplementary material for: From Smartphone Lateral Flow Immunoassay Screening to Direct MS Analysis: Development and Validation of a Semi-Quantitative Direct Analysis in Real-Time Mass Spectrometric (DART-MS) Approach to the Analysis of Deoxynivalenol
Source: Sensors (Basel). 2021 Mar 7;21(5):1861. doi: 10.3390/s21051861 (PMC7962121; doi:10.3390/s21051861)
Supplement: Supplementary file 1 [file sensors-21-01861-s001.pdf]

Supplementary Material

From smartphone lateral flow immunoassay screening to direct MS analysis: development and validation of a semi-quantitative Direct Analysis in Real Time Mass Spectrometric (DART-MS) approach to the analysis of deoxynivalenol.

Ariadni Geballa-Koukoula [1,*], Arjen Gerssen [1] and Michel W.F. Nielen [1,2]

[1] Wageningen Food Safety Research, Wageningen University and Research, P.O. Box 230, 6700 AE Wageningen, The Netherlands; ariadni.geballakoukoula@wur.nl; arjen.gerssen@wur.nl; michel.nielen@wur.nl

[2] Laboratory of Organic Chemistry, Wageningen University, Stippeneng 4, 6708 WE Wageningen, The Netherlands

[*] Correspondence: ariadni.geballakoukoula@wur.nl

1. DART-QqQ-MS/MS method development

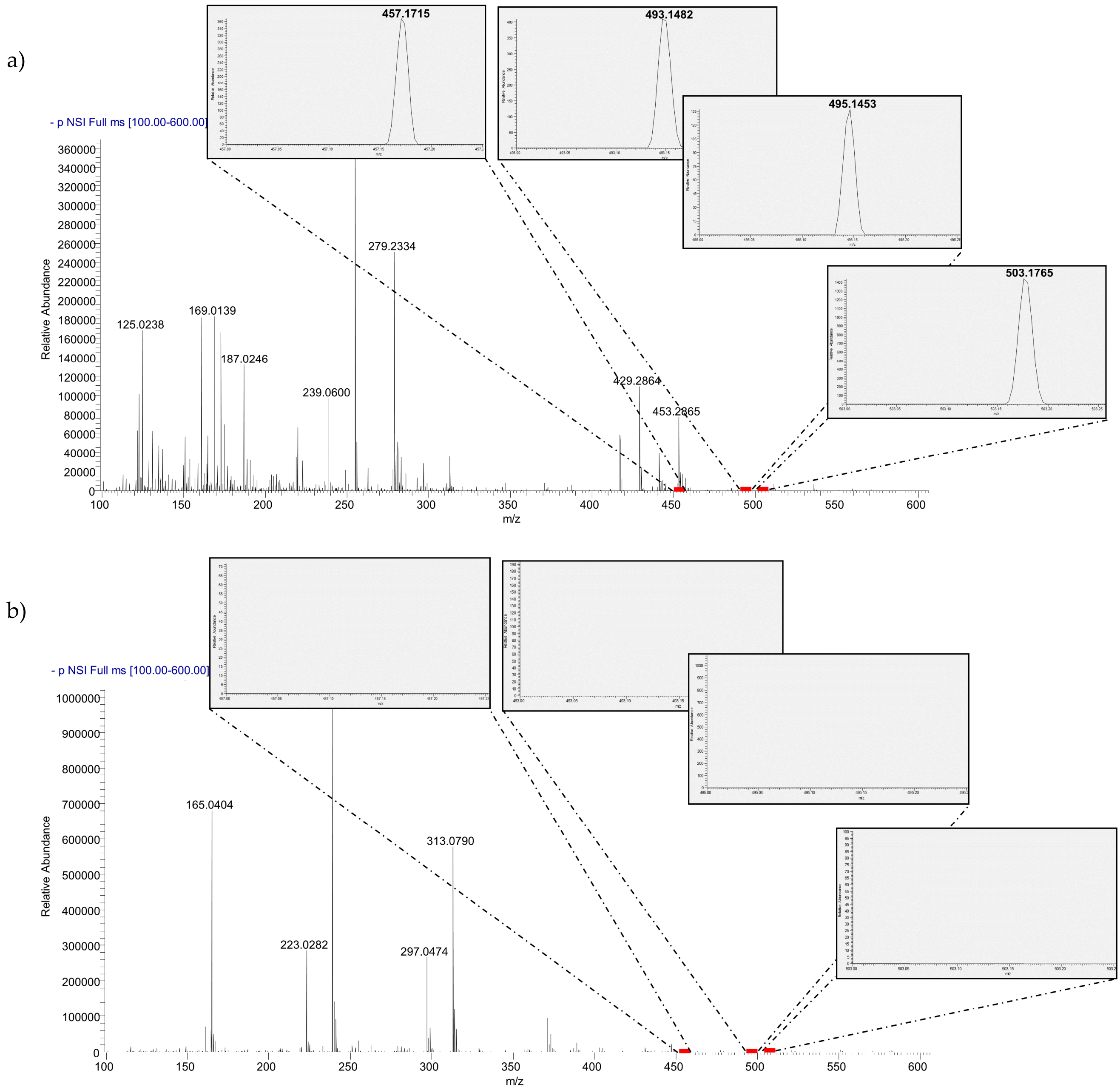

Figure S1. Ionization in DART-Orbitrap MS negative ion mode, full scan *m/z* 100-600, of (a) DON3G in MeOH/NH_3 2% *v/v* and (b) blank MeOH/NH_3 2% *v/v* solution. Detection of the characteristic deprotonated ion and adduct ions can be seen in the inserted zoomed-in mass spectra for *m/z* 457.1715 $[M-H]^-$, *m/z* 493.1482/495.1453 $[M+Cl]^-$ and *m/z* 503.1765 $[M+FA-H]^-$. Note: formic acid (FA) and chloride are impurities in the solvents and/or the MS background.

(a)

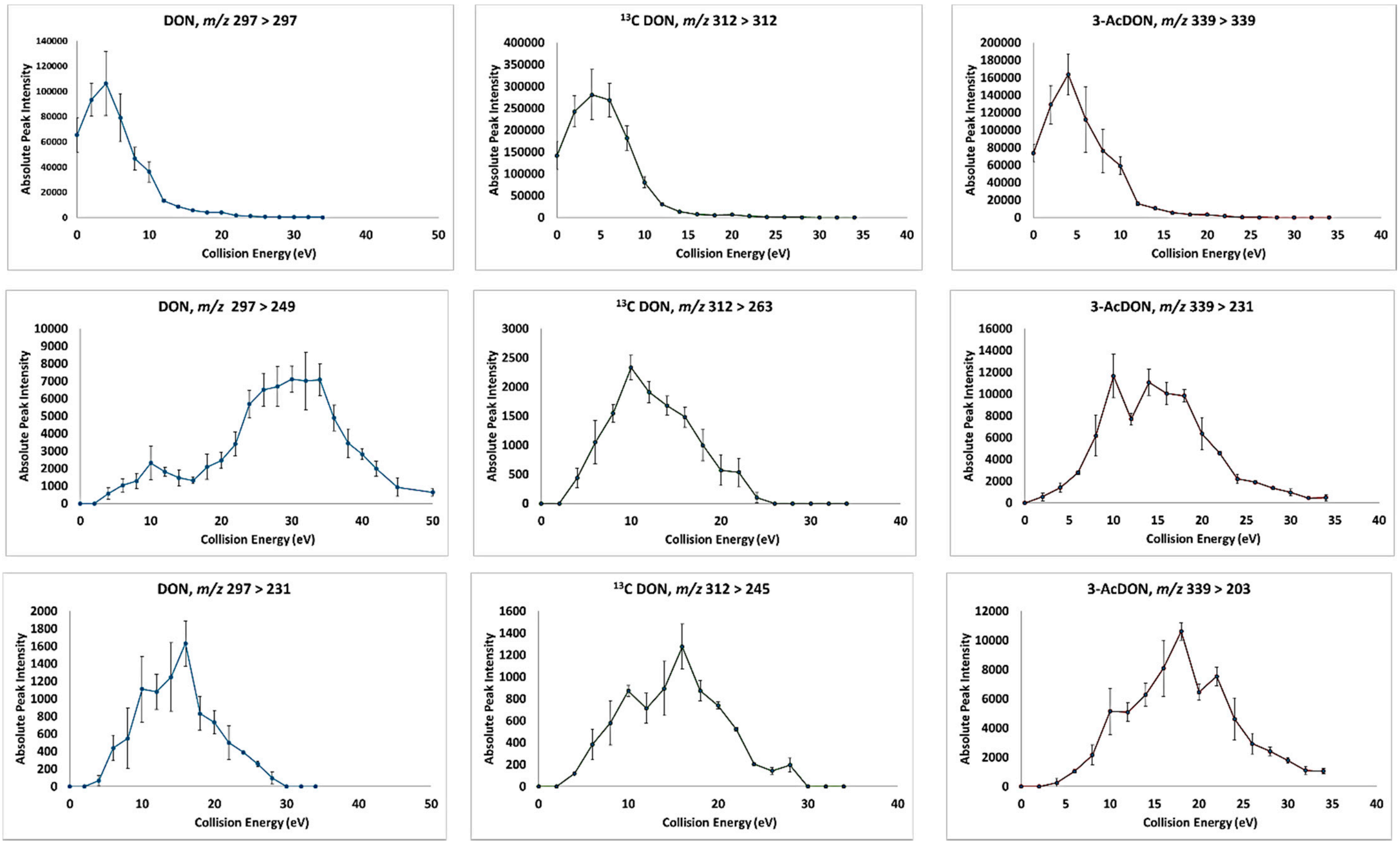

(b)

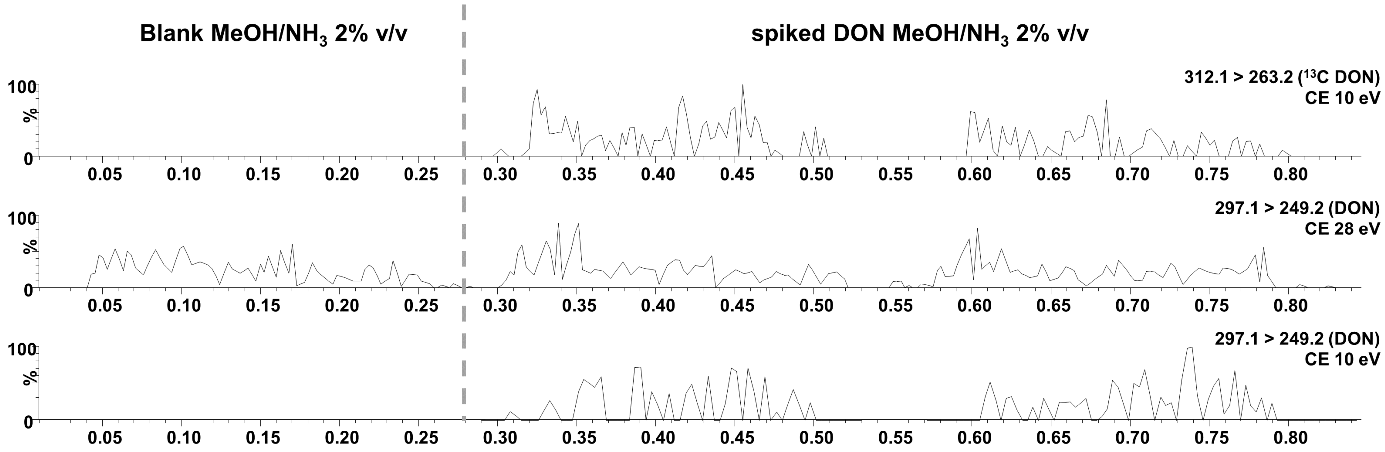

Figure S2. (a) MS/MS fragmentation optimization of DON (blue line), $^{13}C_{15}$ DON (green line), and 3-AcDON (red line) in DART-QqQ-MS/MS. The graph depicts the absolute peak height intensity of the ion transitions in MRM mode versus the collision energy. Measurements were performed in duplicate, and the standard deviation of the duplicate measurement is shown by the error bars. (b) DART-QqQ-MS/MS chronograms of the selected ion transitions for DON and the internal standard, $^{13}C_{15}$ DON. At first, a blank MeOH/NH_3 2% *v/v* solution is being analyzed, followed by a duplicate sampling of a spiked DON plus $^{13}C_{15}$ DON 60 ng/mL in MeOH/NH_3 2% *v/v*, at two different collision energy (CE) settings, demonstrating the inability of the wrong CE setting at 28 eV to differentiate between blank and spiked sample.

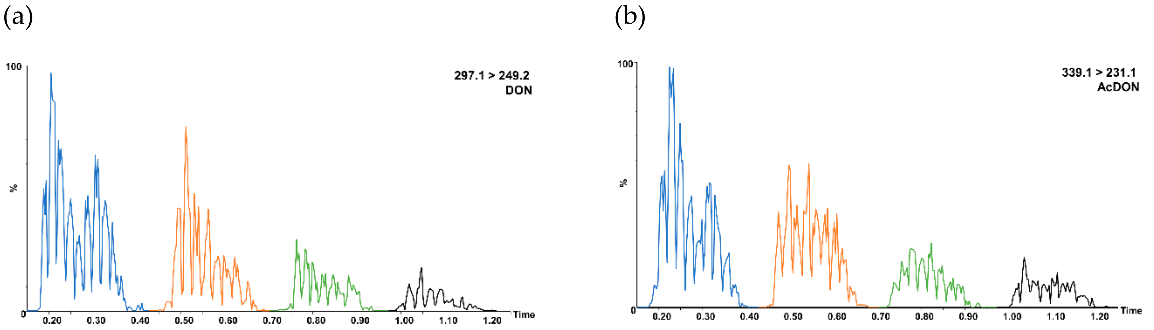

Figure S3. Chronograms of positive ionization DART-MS/MS of different dissociation solution compositions of 60 ng/mL (a) *m/z* 297.1 > 249.2 for DON, and (b) *m/z* 339.1 > 231.1 for AcDON. MeOH/NH_3 2% *v/v* (blue), ACN/NH_3 2% *v/v* (orange), MeOH/HCOOH 2% *v/v* (green), ACN/HCOOH 2% *v/v* (black).

2. Method Validation

2.1. Linearity range of ID-LFIA/DART-QqQ-MS/MS

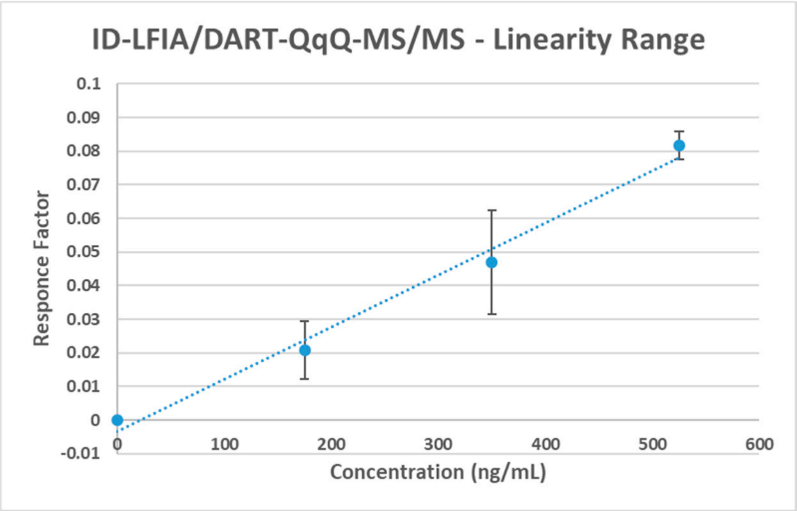

Figure S4. Linearity curve of DON retrieved from ID-LFIA-DART-QqQ-MS/MS analysis of wheat spiked samples on three different levels, namely 175 , 350 and 525 ng/mL corresponding to 0-1.5 times the ML of 1.75 mg/kg of DON in unprocessed durum wheat

2.2. Monoclonal Antibody Specificity

For the specificity testing Surface Plasmon Resonance (SPR) measurements were performed in a Biacore 3000 (GE Healthcare, Uppsala, Sweden) using a carboxymethylated dextran-coated gold chip (CM5) (GE Healthcare, Uppsala, Sweden) with a conjugate of DON with BSA (DON-BSA) (Aokin AG, Berlin, Germany) immobilized on the surface. HBS-EP was used as a running buffer at 5 µL/min flow rate, and 20 µL was injected of a 1:1 mixture of anti-DON mAb 0.3 mg/mL: mycotoxin 0.3 mg/mL. When the mycotoxin analyte interacts with the antibodies in the mixture, then no signal is observed since the mAbs are occupied and cannot bind to the immobilized DON-BSA conjugate. Contrary, when the antibodies do not interact with the analyte in the sample, an increase in the SPR signal is observed because of the antibodies' binding with the DON-BSA immobilized on the chip. After the binding

occurs, regeneration of the chip is performed, with NaOH 25mM. The results demonstrated antibody-toxin interaction with the toxins: DON, AcDON, DON3G, nivalenol. No binding was observed with other mycotoxins that often occur in wheat: fumonisin B2, T-2 toxin, and zearalenone.

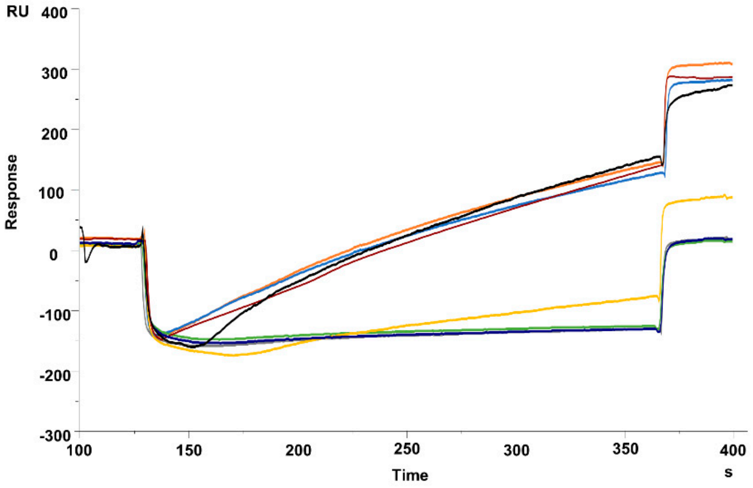

Figure S5. Overlay SPR sensorgrams from the competitive inhibition measurements of *Fumonisin* sp. toxins: DON (dark blue), AcDON (grey), DON3G (yellow), nivalenol (green), fumonisin B2 (light blue), T-2 toxin (dark red), zearalenone (orange) and blank (black).
